# Terrestrial health applications of visual assessment technology and machine learning in spaceflight associated neuro-ocular syndrome

**DOI:** 10.1038/s41526-022-00222-7

**Published:** 2022-08-25

**Authors:** Joshua Ong, Alireza Tavakkoli, Nasif Zaman, Sharif Amit Kamran, Ethan Waisberg, Nikhil Gautam, Andrew G. Lee

**Affiliations:** 1 University of Pittsburgh School of Medicine, Pittsburgh, PA USA; 2 Human-Machine Perception Laboratory, Department of Computer Science and Engineering, University of Nevada, Reno, Reno, NV USA; 3 University College Dublin School of Medicine, Belfield, Dublin, Ireland; 4 Department of Computer Science, Rice University, Houston, TX USA; 5 Center for Space Medicine, Baylor College of Medicine, Houston, TX USA; 6 Department of Ophthalmology, Blanton Eye Institute, Houston Methodist Hospital, Houston, TX USA; 7 The Houston Methodist Research Institute, Houston Methodist Hospital, Houston, TX USA; 8 Departments of Ophthalmology, Neurology, and Neurosurgery, Weill Cornell Medicine, New York, NY USA; 9 Department of Ophthalmology, University of Texas Medical Branch, Galveston, TX USA; 10 University of Texas MD Anderson Cancer Center, Houston, TX USA; 11 Texas A&M College of Medicine, Bryan, TX USA; 12 Department of Ophthalmology, The University of Iowa Hospitals and Clinics, Iowa City, IA USA

**Keywords:** Eye manifestations, Disease prevention

## Abstract

The neuro-ocular effects of long-duration spaceflight have been termed Spaceflight Associated Neuro-Ocular Syndrome (SANS) and are a potential challenge for future, human space exploration. The underlying pathogenesis of SANS remains ill-defined, but several emerging translational applications of terrestrial head-mounted, visual assessment technology and machine learning frameworks are being studied for potential use in SANS. To develop such technology requires close consideration of the spaceflight environment which is limited in medical resources and imaging modalities. This austere environment necessitates the utilization of low mass, low footprint technology to build a visual assessment system that is comprehensive, accessible, and efficient. In this paper, we discuss the unique considerations for developing this technology for SANS and translational applications on Earth. Several key limitations observed in the austere spaceflight environment share similarities to barriers to care for underserved areas on Earth. We discuss common terrestrial ophthalmic diseases and how machine learning and visual assessment technology for SANS can help increase screening for early intervention. The foundational developments with this novel system may help protect the visual health of both astronauts and individuals on Earth.

## Introduction

Spaceflight Associated Neuro-Ocular Syndrome (SANS) refers to a constellation of neurologic and ocular, clinical and imaging findings observed in astronauts following long-duration spaceflight (LDSF). These findings include optic disc edema, posterior globe flattening, total and retinal nerve layer thickening, optic nerve sheath distension, chorioretinal folds, retinal cotton wool spots, and hyperopic refractive shift^1–3^. Some of the findings in SANS (e.g., globe flattening and refractive error) can persist for years after returning to Earth^1,3–5^. The National Aeronautics and Space Administration (NASA) has been closely documenting these findings and has assigned SANS an elevated “Likelihood and Consequence” rating largely based on the large uncertainty surrounding the impact it can have on astronaut health and performance. This rating indicates that improved characterization and mitigation of SANS is critical for future planetary missions^2^.

**Fig. 1:**
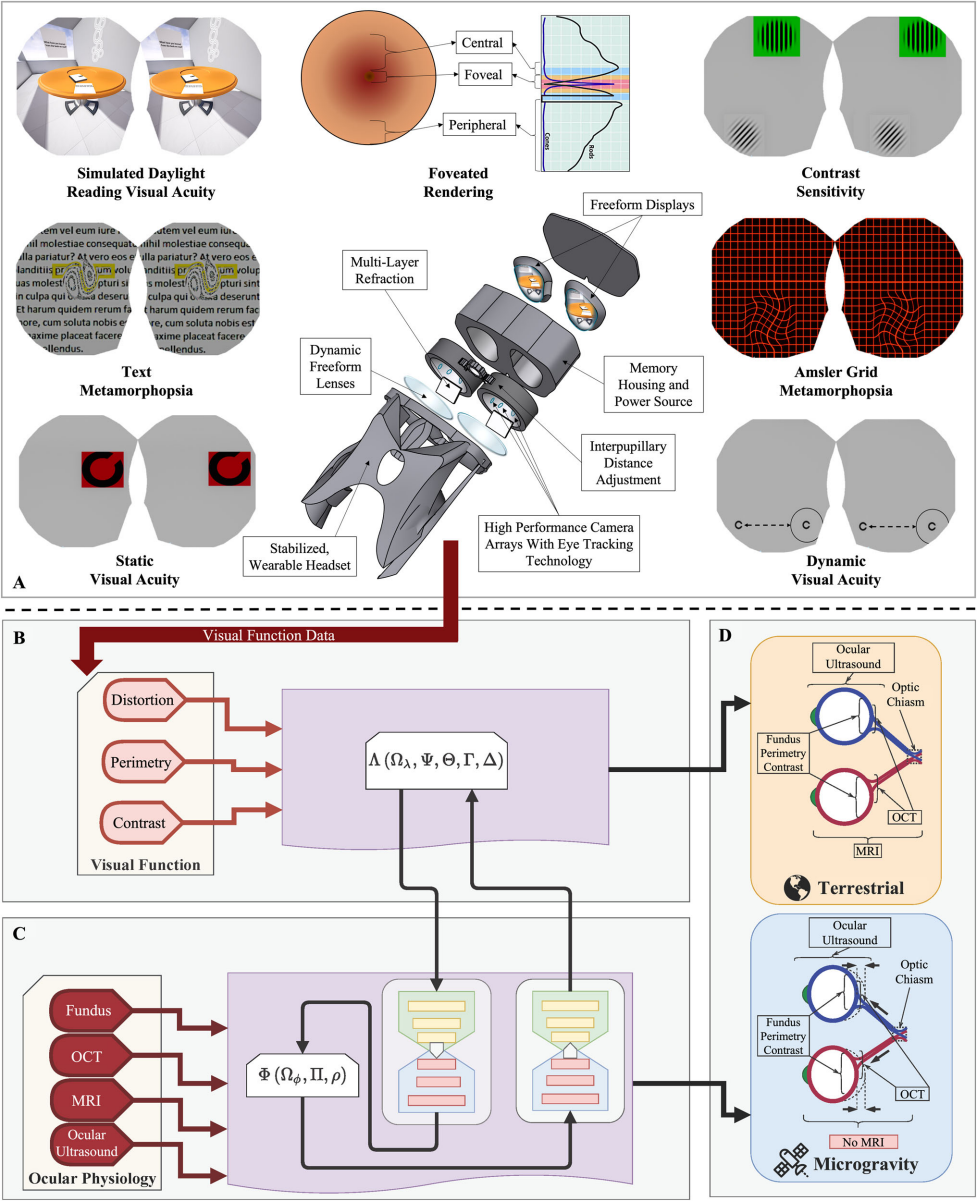
Roadmap in novel monitoring and researching the pathogenesis of spaceflight associated neuro-ocular syndrome (SANS) enabled by multi-modal visual assessment technology and machine learning. Data enabling study of etiology and pathogenesis of SANS is built upon two domains of data: visual function (**A**, **B**) and ocular structure (**C**). Two main research avenues must be established. **A**, **B** Novel multi-modal visual assessment with virtual reality technology to quantify parameters of visual function (Λ) changes caused by SANS-specific neuro-ophthalmic structural changes. **C** Novel techniques to establish shared and complimentary representations (Φ) of both the structure changes and the changes made to the parameters of the visual function due to SANS. **D** These novel parametric functional representations and the accompanying mappings between the visual function and ocular structure can provide a comprehensive and whole some battery of assessments capable of measuring the impact from each domain (e.g., structural changes) on the other (e.g., visual function symptoms). These techniques should be deployed and tested both terrestrially and under microgravity conditions to ensure their reliability, specificity, and sensitivity for both terrestrial and spaceflight applications. Illustration by Joshua Ong, Nasif Zaman, Sharif Kamran, and Alireza Tavakkoli.

Although the exact pathophysiology for SANS is not completely understood, close pre-, in-, and post-flight monitoring of astronauts is on-going. NASA has funded the development of a compact virtual reality (VR) device integrated with multi-modal visual assessments, computational mapping tools, and machine learning frameworks to closely assess ocular structure and functional changes during LDSF^6^. The multi-modal VR-based visual assessments include visual acuity, contrast sensitivity, dynamic visual acuity, eye-tracking technology, and metamorphopsia assessment^7^. The fusion of VR-based visual assessments and machine learning techniques with structural changes seen on imaging will be required to establish a comprehensive representation of the neuro-ophthalmic structural changes and the symptoms produced by SANS (Fig. 1). The technology is adapted for the limitations of future planetary travel, including limited time for medical testing, stringent weight limits for medical equipment^8^, and reduced communication with terrestrial healthcare experts^9,10^. Parallel technology developments may also find use on Earth for individuals with low access to direct face-to-face eye care. Therefore, these technological innovations including detection and monitoring technology may be adapted to address longstanding barriers to care for terrestrial, vision-threatening ophthalmic diseases. As evidenced by NASA’s *SpinOff* publication, advancing the frontier of space exploration often revolutionizes technology for life on Earth^11^. In this article, we discuss this novel medical technology in its relation to SANS and how it can be applied to prevent irreversible vision loss for low-resource areas on Earth.

## Proposed pathophysiology of SANS

Multiple hypotheses on the pathogenesis of SANS have emerged since the initial description of SANS findings by Mader et al.^3^ Initially termed visual impairment and intracranial pressure (VIIP), SANS has been hypothesized to be due to elevated intracranial pressure (ICP) due to the cephalad fluid shift seen during microgravity^1^. During LDSF, there is a loss in hydrostatic pressure that produces an upward shift of fluid, possibly leading to cerebral venous congestion and elevated ICP. These findings may lead to optic disc edema (ODE) and vision impairment similarly seen in terrestrial idiopathic intracranial hypertension (IIH)^12^. However, SANS is not accompanied with other classic signs of IIH such as pulsatile tinnitus and severe headache. While several astronauts with SANS have had slightly elevated post-flight lumbar puncture opening pressures, other astronauts with SANS have also demonstrated normal post-flight opening pressures^1^. In addition, most IIH patients present with symmetrical ODE whereas more than half of the astronauts with SANS reported by Mader et al. had either unilateral or asymmetric ODE^1,3^ although this finding may reflect the limitations of small sample size and subclinical but bilateral findings in SANS. Further observation and longer durations during spaceflight may give additional insight into the ODE presentation in SANS. These initial reports suggested that SANS may not solely be due to elevated ICP, leading to the name change from VIIP to SANS in 2017^2^.

Another potential hypothesis for SANS revolves around the ocular glymphatic system, a paravascular transport system at the optic nerve (ON)^13^. Recent literature has shown that the biomolecular composition of cerebrospinal fluid (CSF) within the ON sheath (ONS) can differ from the CSF in the spinal cord, suggesting that CSF pressure and composition may differ between various CSF compartments^14^. Wostyn et al. proposed that SANS may be due to a microgravity-induced compartmentalization of CSF within the ONS due to a one-way valve mechanism in the glymphatic system, thus leading to elevated pressures in the ONS while displaying normal post-flight opening pressures^1,13^. Galdamez et al. postulates that microgravity-induced cerebral venous stasis may contribute towards ODE in SANS. Due to cephalad fluid shifts, this stasis may induce insufficient adenosine triphosphate (ATP) generation, thus inhibiting the Na^+^/K^+^ ATPase pump with subsequent edema at the ON head^15^. Strangman et al. hypothesizes that increased cerebral blood volume pulsatility during LDSF may lead to vascular and ocular structural remodeling, potentially explaining the persistence of SANS findings after returning to Earth^16^.

**Fig. 2:**
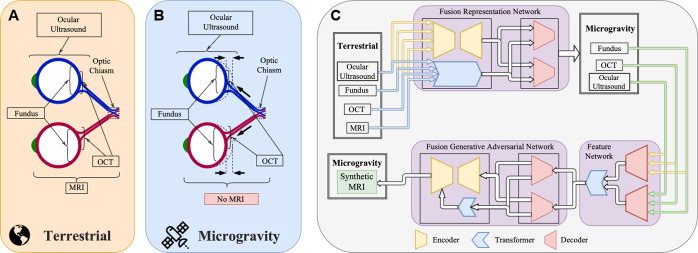
Leveraging artificial intelligence (AI) to study the pathophysiology behind SANS. **A** Terrestrial mechanism such as optical coherence tomography (OCT), orbital ultrasound (OU), fundus photography, and magnetic resonance imaging (MRI) perform various measurements on the structure of the human visual pathways. **B** Post-flight imaging has shown that shifts of the brain and the optic chiasm, among other things, produce ocular structural changes such as globe flattening, choroidal folds, and optic disc edema. Unfortunately, some imaging modalities such as MRI are not conducive to be deployed in-flight. **C** Sophisticated artificial intelligence (AI) techniques such as generative adversarial networks (GANs) have shown promise in their ability to fuse information from multiple data modalities to produce effective representation of the data shared across these modalities. Encoders take encodes from the data domain into the latent/feature space, decoders decode from the latent/feature space back into the data space, and transformers aids with fusion and incorporating temporal and spatial correlations in the data. Inspired by these advances, GAN architectures hold the key in establishing a fusion of representative features among various modalities pertinent to SANS in order to produce imaging data unavailable in-flight. This provides a new era in studying SANS and the risk of its progression.

The proposed pathogenesis of SANS plays a critical role in the development of novel monitoring technology. Post-flight magnetic resonance imaging (MRI) in LDSF astronauts have shown to have an upward shift of the brain and optic chiasm^17^. Shinojima et al. hypothesizes that the mechanical upward brain shift “pulls” the optic nerve posteriorly which produces an anterior counterforce of the dura on the posterior globe, leading to the globe flattening seen in SANS^17,18^. Unfortunately, an MRI scanner is not available onboard the ISS and MRI in SANS is limited to pre- and post-flight testing. Marshall-Goebel et al. discussed the limitations in data for understanding SANS with an emphasis on brain physiology^19^. As MRI data from SANS is collected post-mission, the findings may not be fully representative of the physiological changes that occur during spaceflight^19^. This limitation in data may be a barrier in further understanding SANS and moving towards a specific hypothesis. Direct ICP monitoring in-flight is also not available. From a machine learning perspective, this lack of data in the space environment is a significant barrier to training and validating future models for deployment. To further investigate SANS pathogenesis and build more accurate machine learning models, the use of generative adversarial networks, a powerful machine learning framework, may serve to address several of these limitations. Generative adversarial networks allow for artificial image reconstruction from available modalities, which can be designed to incorporate in-flight imaging to generate a synthetic orbit MRI to monitor globe flattening progression (Fig. 2). Generative adversarial networks may also generate synthetic data that allows for machine learning models to train and become more accurate^20^. This powerful model and its use for SANS will be further elaborated in a subsequent section. While multiple hypotheses have emerged, the true pathogenesis of SANS may be multi-factorial and these hypotheses allow for directed technology development for SANS. The following section describes the development of visual assessment technology that allows for close detection of SANS during LDSF.

## Multi-modal visual assessment technology for spaceflight associated neuro-ocular syndrome

The space environment represents the epitome of resource efficiency. Missions onboard the ISS must be optimized based on various factors including crew number, schedule, equipment weight, size, setup time, usage time, and power consumption^8^. Specific ocular structure imaging technologies such as the fundoscopy, OCT, and ocular ultrasound exist onboard the ISS^1,8,21^. These technologies have been instrumental in understanding SANS. Alongside imaging modalities, visual function assessments such as visual acuity and contrast sensitivity may also help to monitor the clinical and functional outcomes of SANS. To improve SANS monitoring, NASA has funded the mapping of a framework to detect subtle variations in ocular structure utilizing visual function data along with previous imaging data from astronauts^6^. The goal is to augment and analyze indirect, accessible indications (e.g., visual function) of SANS to predict more direct indications (e.g., imaging). For example, subtle hyperopic shifts measured using visual acuity variation may indicate globe flattening and optical axial length changes seen after LDSF^1^. In this section, we discuss various considerations for building a comprehensive visual assessment system for spaceflight that can also be leveraged for low access-to-care areas on Earth.

**Fig. 3:**
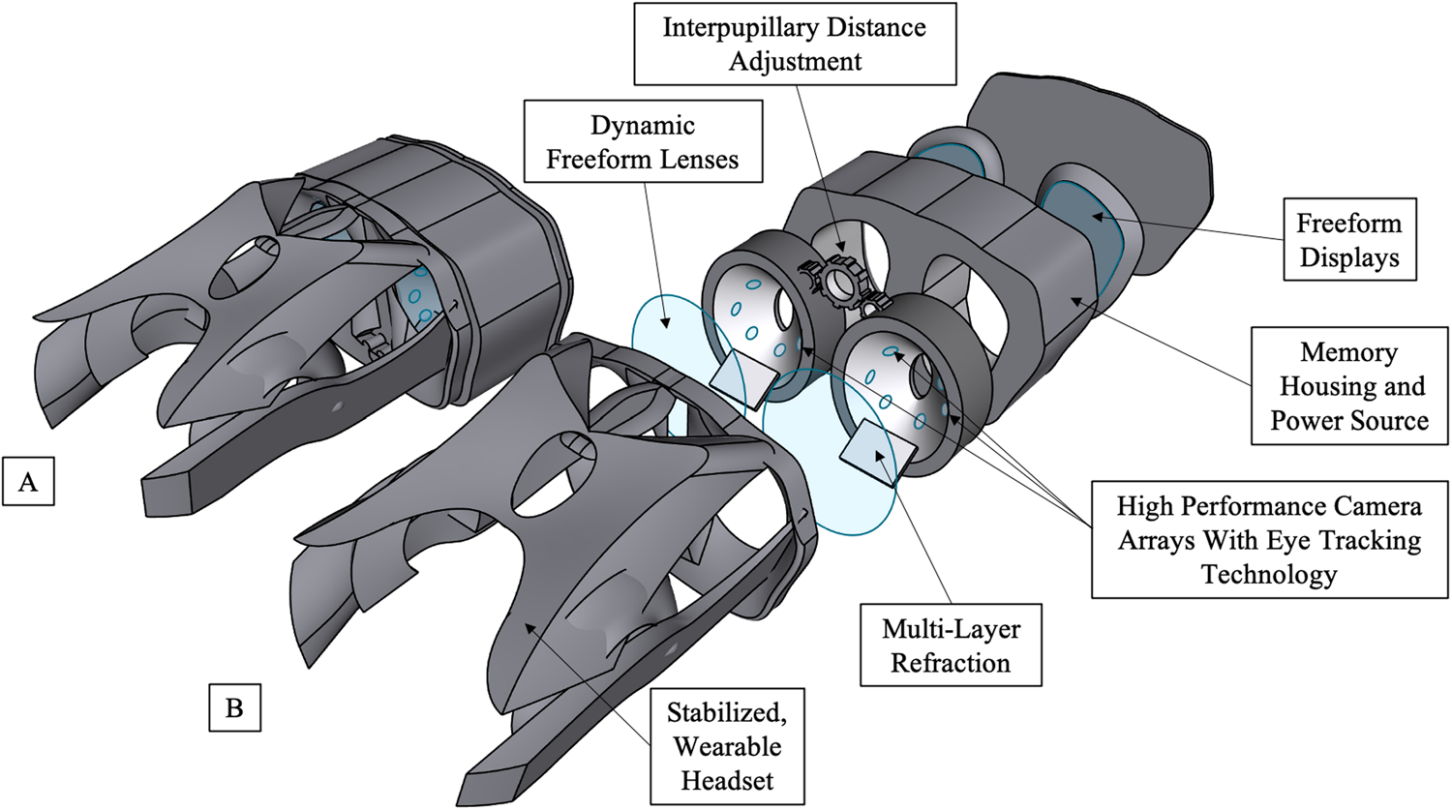
Wearable Multi-modal Visual Assessment System Design. Computer-aided design model of the multi-modal visual assessment virtual reality hardware device with dynamic freeform lenses, multi-layer refraction, eye-tracking camera technology, and freeform displays. Design by Nasif Zaman, Joshua Ong, and Alireza Tavakkoli.

Most modern terrestrial ocular imaging display systems can be modified for LDSF. The visual function tests currently available onboard the ISS use laptops to deliver visual stimuli and measure performance^8^. Head-mounted display technology, which has already been utilized during spaceflight, may be able to further optimize visual functional testing^22^. This technology can decrease testing times, tighten control over dichoptic stimuli presentation, and eliminate the influence of external illumination^23,24^. Virtual reality (VR) technology now offers eye tracking that can help measure saccade, adaptation, and retinal loci^25,26^, greatly increasing the breadth of information available to understand SANS mechanisms. With these considerations in mind, SANS research currently includes building a compact, VR-based system that efficiently measures visual acuity, color and contrast sensitivity, and visual distortions across the visual field with multiple sessions to develop a robust model of the astronauts’ vision throughout longitudinal missions (Fig. 3)^6^.

The efficient properties that make this multi-modal assessment system useful onboard the ISS also make it valuable for underserved areas on Earth. A portable, self-guided screening tool can be stationed in rural areas with limited access to eye care. The low operating cost of the system may lead to increased compliance and frequency in testing as suggested by other VR eye care studies^24,27^. Baseline evaluations can be established in the earlier test phases and the gradual aggregation of functional data would lead to a reliable model of the individual’s ocular health. As we discuss in later sections, early detection and intervention in common ophthalmic diseases such as glaucoma is critical for preventing vision loss^28^.

## Comprehensive visual assessments during spaceflight

Decreased visual function in astronauts might lead to loss of productivity during missions, thus, close monitoring is of utmost importance. Currently onboard the ISS, astronauts undergo many routine functional visual assessments (e.g., visual acuity, Amsler grid test). Contrast sensitivity testing is also available^8^. These tests have well-established terrestrial applications^29^. For optimal monitoring, these visual assessments may benefit from consistent distancing and illumination calibration to reduce the subjectivity of the tests. These objectives may be achieved through virtual reality (VR) head-mounted systems. The laptop screen-based tests available onboard the ISS may be repurposed for an immersive experience with this technology. Additionally, if all visual function tests are delivered using one VR device, it will be possible to make inference on other tests once a session is recorded. Specifically for SANS monitoring, it is important to identify any subtle perceptual impact so that countermeasures can be designed. Intelligent delivery of stimuli under various conditions would help identify subtle perceptual loss.

**Fig. 4:**
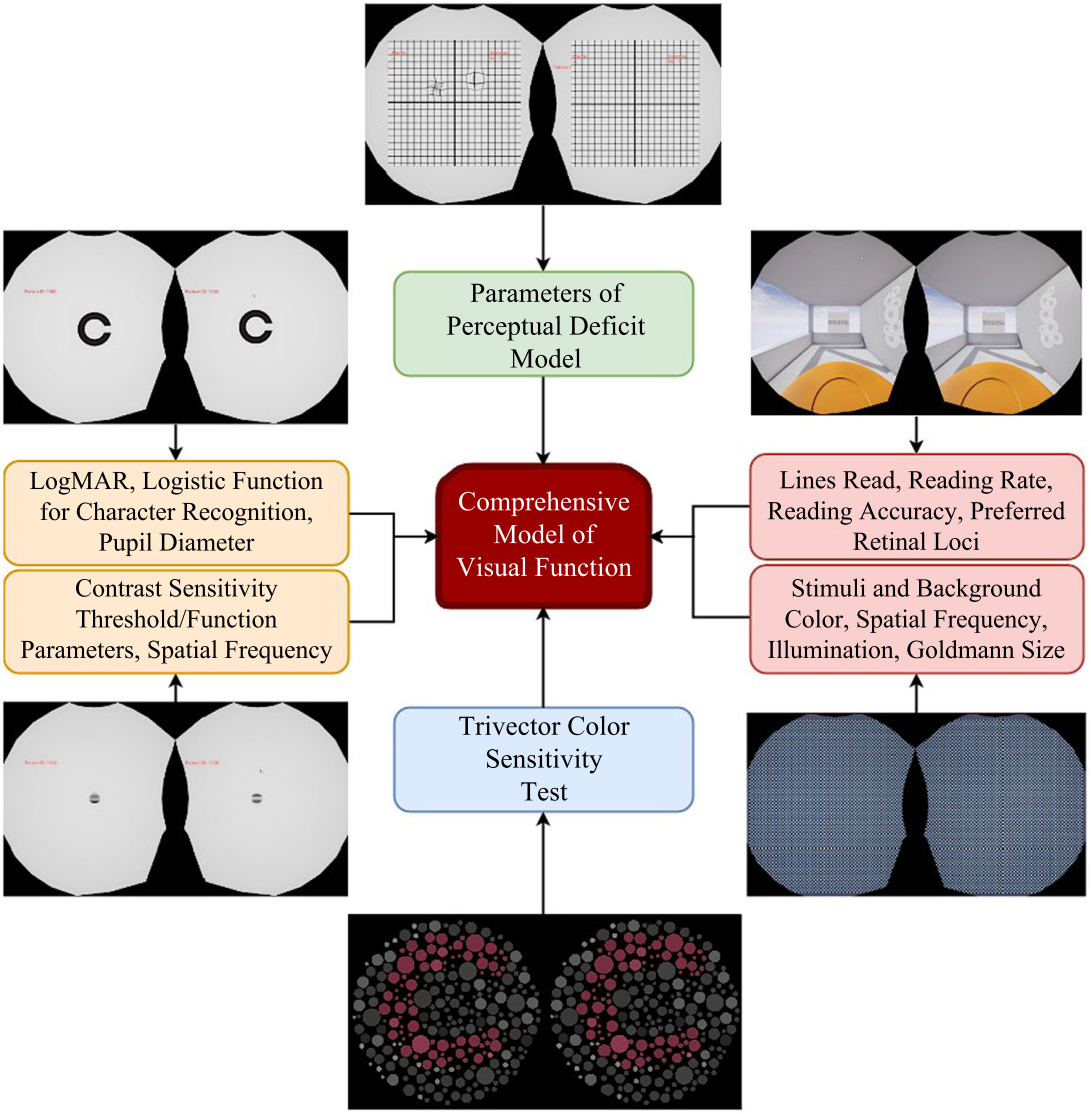
Framework for a Comprehensive Model of Visual Function. Multi-modal visual assessments for building a comprehensive model of visual function for SANS monitoring. These assessments include visual acuity evaluation, trivector color sensitivity test, reading accuracy, and contrast sensitivity threshold/function parameters.

Optic disc edema, globe flattening, nerve fiber layer thickening, and choroidal folds are common imaging findings in SANS^1^. While it is important to monitor SANS, frequently repeating these imaging tests to would consume a significant portion of mission time. Therefore, quick sessions of different visual function tests are being considered to continually track the different aspects of SANS symptoms. This can be achieved by mapping visual functional data with imaging data using pre-existing astronaut data as well as head-down tilt bed rest, an analog for SANS^30^. Several primary tests will be important for this system including visual acuity, contrast sensitivity, Amsler grid, and visual fields (Fig. 4). These assessments can be linked to specific SANS findings that parallel terrestrial ocular relationships such as contrast sensitivity and retinal nerve fiber layer thickening^31^. In addition, these visual function tests may be able to further characterize any deficiencies in SANS by providing additional visual assessment tests. In the terrestrial pathologies section, we highlight how many of these visual assessments can be utilized to screen and monitor common ophthalmic pathologies.

Lastly, the ideal implementation of this system would be to initially establish a baseline for astronauts on Earth. Specific modeling and stimulus presentation in a completely controlled immersive environment would provide a complementary metric. A Bayesian approach where each session updates the parameters based on all previous sessions make it possible to continually track the status of the important visual parameters^32^. The implementation of such a system, in parallel with intelligent algorithms onboard to decipher imaging and assessment information, can greatly improve SANS monitoring for future spaceflight.

## Machine learning in spaceflight associated neuro-ocular syndrome (SANS)

When anticipating limitations for deep space exploration, the delayed communication to imaging specialists and prolonged absence of terrestrial-only imaging are critical aspects to consider for SANS. Currently, in-flight imaging modalities can be employed to computationally extract objective information. However, detailed interpretation often requires terrestrial specialists to analyze and distinguish SANS findings^1^. For future space exploration, the communication bandwidth may be insufficient for effective exchange of high-quality images and communication between terrestrial healthcare providers and astronauts^33^. In addition, there are restrictions to medical devices that have been instrumental to understanding SANS such as MRI^17,18^. To optimize the utility of in-flight imaging and closely monitor SANS, artificial intelligence (AI) frameworks serve as promising solutions to address many limitations anticipated during planetary missions. In this section, we cover AI machine learning techniques, including generative adversarial networks and unsupervised learning, that are being developed to optimize the detection of SANS and address the issue of limited imaging modalities. These techniques are concurrently developed to pair with the visual assessment VR technology, providing a powerful diagnostic tool for both astronauts and communities with limited access to care.

Machine learning systems often require large amounts of data for effective algorithm training. With a large emphasis on imaging, the field of ophthalmology is well-suited for this technology; this is evidenced by IDx-DR, an AI diabetic retinopathy diagnostic system and the first AI device approved for clinical use by the Food and Drug Administration^34^. Machine learning in ophthalmology has been successfully applied to various modalities, such as applying machine learning with fundoscopy and OCT to detect glaucoma and segment retinal layers^35^. Given that these imaging modalities are also onboard the ISS for ocular health monitoring, machine learning serves as promising asset for understanding SANS.

A large challenge to developing machine learning applications for human spaceflight is the severe limitation in data for training and validating machine learning models. Less than 600 individuals have flow to space across a period of multiple decades^36^. In-flight ophthalmic imaging modalities have also differed over these decades which adds another layer of dataset insufficiency. When machine learning models are trained poorly with insufficient data, the outcomes of the model when exposed to novel, external scenarios may be inaccurate. Validation of the model is also an important step in understanding the accuracy of the machine learning framework, which must also have a large enough dataset for reliable assessment. Analyses of machine learning algorithms for terrestrial diabetic retinopathy have strongly demonstrated the need for rigorous testing on real-world data prior to integration into clinical use^37^. At the current trajectory, machine learning models built just upon astronaut data may not perform at the most optimal level to accurately monitor and assess for anticipated missions in the coming decade. Several considerations/methods may be utilized to address this unique challenge in machine learning for astronaut health. Transfer learning is a neural network technique that has been utilized terrestrially to address the lack of labeled large imaging datasets^38,39^. Transfer learning takes a pre-trained model, typically trained on a much larger labeled dataset, and applies parts of the neural network layers to the new model of interest. This approach of reusing a learned, accurate model is highly effective for building more robust models in scenarios of insufficient or limited datasets for training. Validated, labeled terrestrial models trained on large datasets that have specific features of interests may serve as a proper source model for increasing the robustness of these machine learning frameworks for spaceflight. Another method to circumvent the insufficiency of data is to supplement with terrestrial analog data. Head-down tilt bed rest is a terrestrial analog that mimics the cephalad fluid shifts in spaceflight and has been observed to produce optic disc edema and chorioretinal folds within 60 days^40^. While these bed rest studies do have their own terrestrial limitations (e.g., duration of studies), the relative cost and time compared to spaceflight is much less. Lastly, as commercial spaceflight continues to grow, it is anticipated that more individuals will travel to space at a larger rate. SANS develops after LDSF, thus short-duration spaceflight will likely not yield data. However, as the commercialization of spaceflight continues to rapidly expand, more individuals may be exposed to prolonged periods of microgravity and develop SANS. This increase in data will be helpful for developing machine learning techniques for exploration spaceflight.

**Fig. 5:**
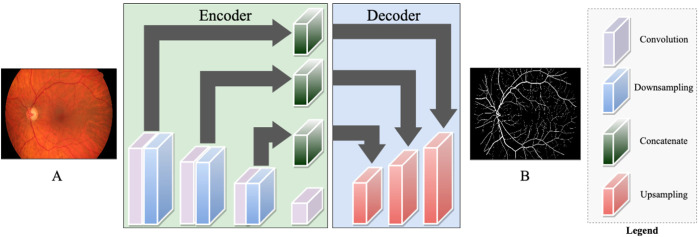
Supervised Machine Learning Approach for Retinal Vessel Segmentation. A supervised approach for retinal vessel segmentation from a retinal fundus image using Auto-encoder architecture. The architecture consists of multiple convolutions, down sampling, up sampling blocks and skip connections in between using concatenate layers.

Two highly applied machine learning techniques in ophthalmic imaging are called supervised learning and unsupervised learning. The main difference between these machine learning algorithms is that supervised learning pairs input data with an annotation to train the model end-to-end whereas unsupervised learning only has input data to train the model. Supervised learning techniques include image classification and object detection^41,42^ and unsupervised learning techniques include image reconstruction and image denoising^43,44^. Since fundoscopy and OCT are available on the ISS, terrestrial specialists can relatively quickly employ machine learning techniques to further understand ocular physiology and SANS with in-flight modalities during LDSF. Supervised learning algorithms have demonstrated success in various ophthalmic tasks, including hemorrhage detection, retinal vessel segmentation, and glaucoma localization^45–47^. In Fig. 5, we illustrate a supervised learning auto-encoder architecture for retinal vessel segmentation from a fundus image that can be applied to astronaut fundoscopy (Fig. 5)^48^. Angiographic extraction of the retinal vasculature may play an important role in further understanding SANS. A recent study observed decreased arterial and venous densities in astronaut retinas after six-month missions^49^. The authors conclude that retinal vascular remodeling may represent as a useful biomarker for understanding SANS, and that further research is necessary to better characterize these retinal changes to spaceflight^49^. Deep learning-based retinal segmentation allows for robust evaluation of important biomarkers including branching angles, bifurcations, vessel tortuosity, and artery-vein ratio^50^. By merging this technique with an imaging modality onboard the ISS, in-flight changes in retinal vasculature may be further understood.

**Fig. 6:**
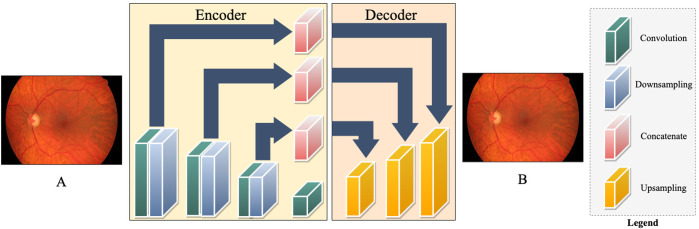
Unsupervised Machine Learning Approach for Retinal Fundus Image Denoising. An unsupervised approach for denoising fundus images using Auto-encoder architecture. The architecture consists of an encoder and decoder network. The encoder consists of convolution followed by downsampling layers, Whereas the decoder consists of upsampling layers. Other than that, it has multiple skin connections in between using concatenate layers.

Unsupervised learning is a valuable technique to address data transfer speeds during spaceflight. The technique is carried out using an auto-encoder for extracting and retaining intricate features. An auto-encoder is made of an encoder and decoder to reduce data dimensions. This machine learning architecture is powerful for medical image denoising, reconstruction, and compression^35,51,52^. During spaceflight, unsupervised learning is effective for SANS by denoising and removing artifacts from limited in-flight imaging to detect subtle changes, as well as compressing key images while maintaining critical features to expedite transmission times to Earth during exploratory missions. As illustrated in Fig. 6, an autoencoder is employed to reconstruct the fundus image, successfully denoising the image in the process (Fig. 6)^48^.

**Fig. 7:**
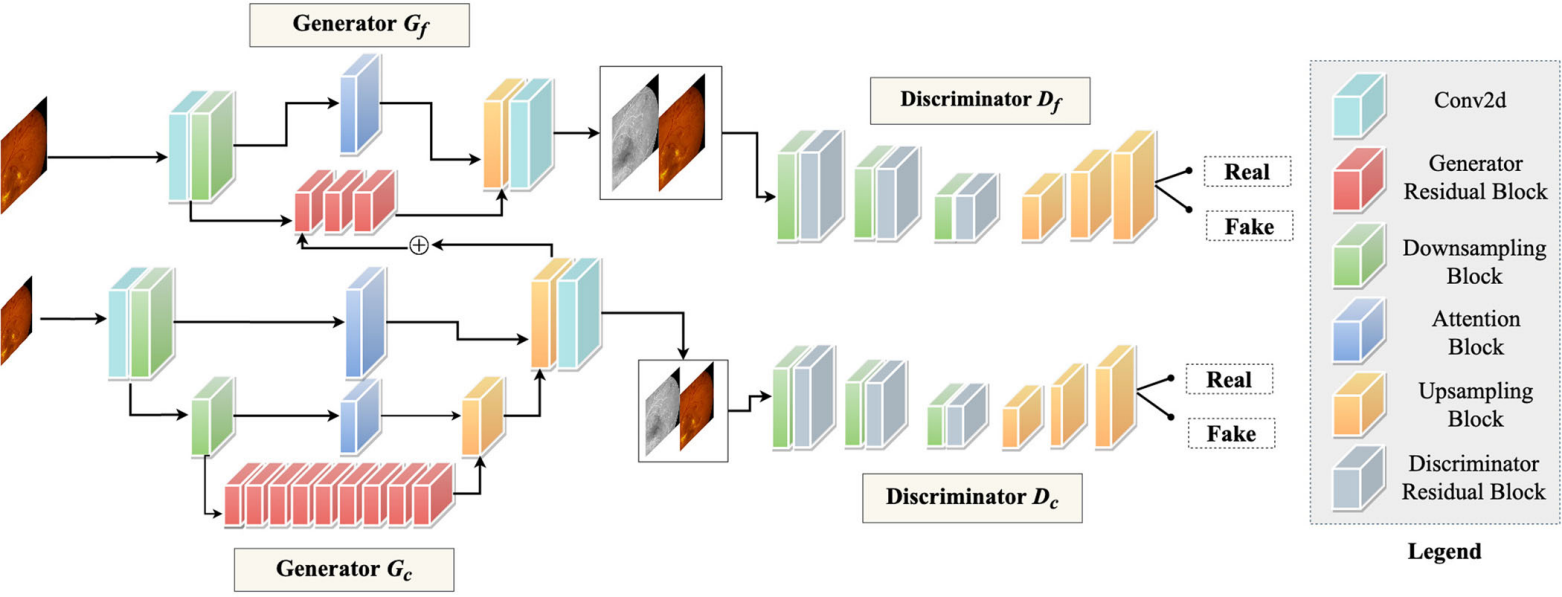
A multi-scale generative network for fluorescein angiography (FA) synthesis from color fundus. The model comprises of two generators and two discriminators for taking in images of different resolutions and scales. There is a feature fusion between the coarse and fine generators, which extracts both global and local features. The discriminators take in pairs of fundus and FA images and dictates if the image pairs are real or fake. The generator only other hand synthesizes FA from retinal fundus images at two different scales.

Since machine learning has been employed terrestrially with fundoscopy and OCT, deploying a similar automated system in ISS is likely to be effective. In SANS, optic disc edema and nerve fiber layer thickening can be detected by incorporating deep learning techniques, and cotton wool spots and globe flattening can be identified and localized using segmentation-based deep learning architectures^53,54^. Unfortunately, imaging modalities such as MRI and fluorescein angiography (FA) are not available on the ISS, which have been instrumental in SANS proposed pathogenesis and confirming choroidal folds after LDSF, respectively^17,18^^,^^55^. This limitation can be addressed by implementing the revolutionary deep learning architecture termed “Generative Adversarial Network” (GAN). GANs can synthesize images from one modality to another, such as generating of FA images from fundus images^56^, and generating fundus autofluorescence from OCT^57,58^, thus, creating an artificial imaging modality. Figure 7 illustrates a multi-scale GAN for FA synthesis from color fundus photographs (Fig. 7)^59^.

GANs learn by playing a min-max “game” between two distinct architectures termed “Generator” and “Discriminator”^60^. The generator attempts to synthesize realistic images of Modality B from Modality A, whereas the discriminator is tasked to distinguish between real and fake modality B images. This is work can be furthered extended to incorporating multiple modalities of images to generate the missing data. For example, GANs can be developed to extract valuable features in fundoscopy, OCT, and ocular ultrasound. In addition, functional information like visual acuity, metamorphopsia, and contrast sensitivity from the multi-modal VR system can be merged into GAN frameworks to optimize the synthesis of an artificial imaging modalities. The GAN output does not have to be a single structured data like MRI, fundus, or OCT; instead, we can have multiple outputs for detecting and localizing underlying conditions like globe flattening, cotton spots, optic disc edema, and choroidal folds. Similar work was done by incorporating retinal nerve fiber layer maps, confocal scanning laser ophthalmoscopy imaging, and enface images to detect glaucomatous visual field defects and predict mean deviation of visual fields from spectral domain-OCT images^61^. The technological innovation of the Hood Glaucoma Report software in OCT allows for precise diagnostic information and optimizes detection of subtle changes in glaucoma^62^. In the future, similar machine learning architectures trained on astronaut and terrestrial analog data can be deployed for detecting SANS and will help monitor disease progression during LDSF in real-time.

Ultimately, machine learning and VR visual assessment technology can address many of the limitations for monitoring SANS during planetary travel. When these limitations are broken down to the core fundamentals, such as limited access to ophthalmic screening and imaging modalities, they closely parallel with limitations seen in low access-to-care areas on Earth. As the number of individuals affected by preventable vision impairment is anticipated to grow by the millions in the coming decades^63,64^, it is imperative to increase access to screening and imaging modalities. Several future considerations for integration of these technologies include in-flight computational capabilities and integrated software to directly feed imaging data into established machine learning models. Further insight into these integration considerations will be more strongly established as the technology progresses. While serving an incredible use for detecting SANS, innovations in machine learning and VR technology can be leveraged to benefit many communities across the world that experience barriers in receiving optimal vision care. In the following section we discuss how these innovations for SANS can help preserve vision and quality of life on Earth.

## Terrestrial ophthalmic diseases and preventable irreversible vision loss

**Fig. 8:**
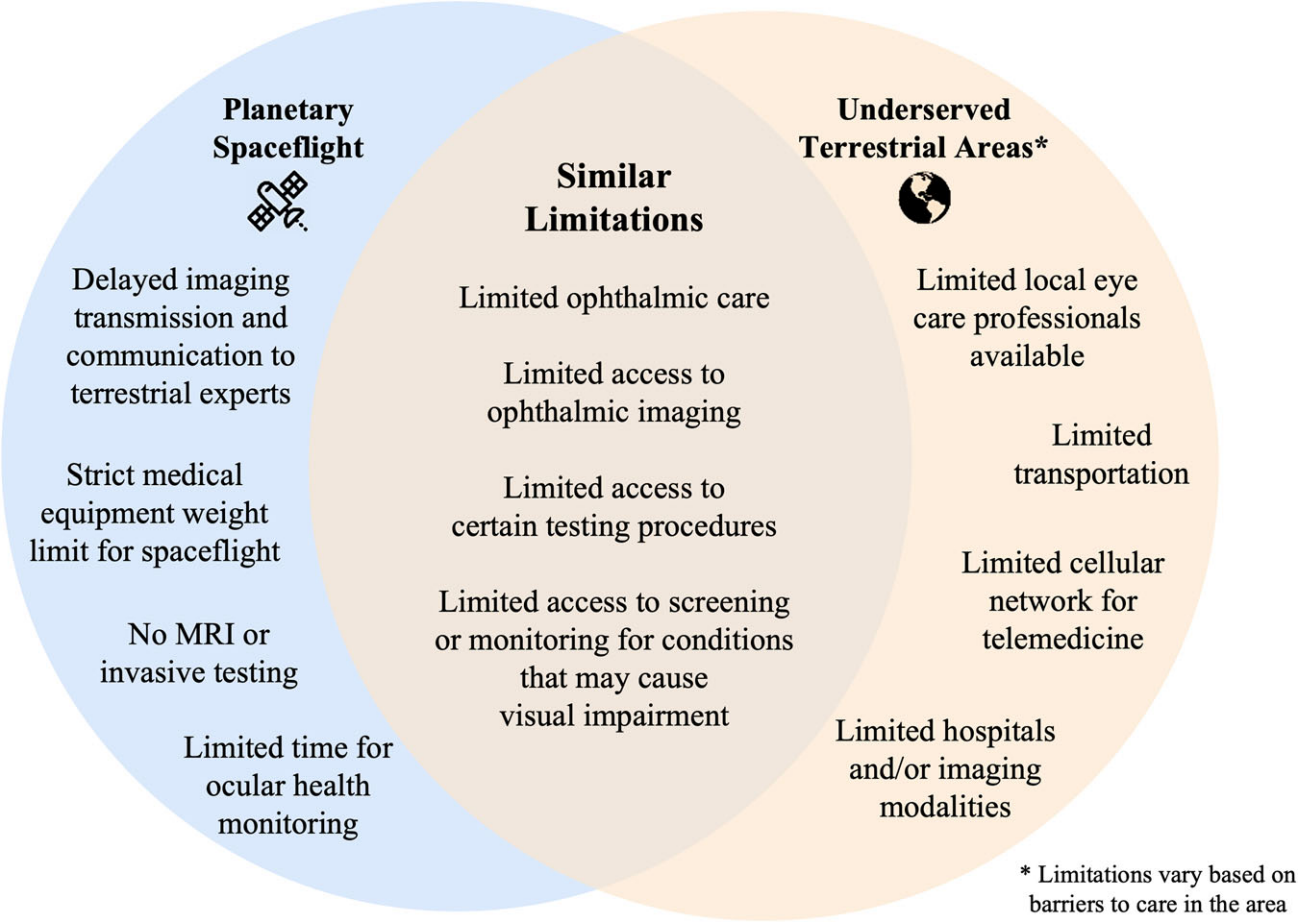
Parallels in the limitations for ophthalmic care seen in spaceflight and in underserved communities on Earth. The spaceflight environment and underserved areas on Earth have overlapping similarities. These parallels include limited ophthalmic care, limited access to ophthalmic imaging, limited access to certain testing procedures, and limited access to screening and monitoring for vision-threatening conditions.

The World Health Organization estimates that over 2.2 billion people have some level of visual impairment with at least 1 billion arising from preventable or unaddressed causes^65^. With larger aging populations, providing widespread, cost-effective solutions to prevent vision loss becomes increasingly critical and novel interventions are needed. The development of the advanced visual assessment technology with machine learning for the austere environment of spaceflight can help attenuate these risks and address longstanding barriers to ophthalmic healthcare on Earth. Several barriers seen on Earth parallel challenges faced during spaceflight (Fig. 8). In this section, we discuss common vision-threatening diseases that may benefit from the multi-modal vision technology and machine learning frameworks being developed for SANS.

Diabetic retinopathy (DR) is one of the leading causes of vision loss in working-age individuals globally^66^. In 2020, approximately 103 million people had DR and this number is expected to reach 224 million by 2040^63,67^. For conditions such as proliferative DR, the outcomes of treatment are highly dependent on the timing of laser treatment, which is optimally administered prior to vision being significantly affected but when high-risk characteristics are present (e.g., microaneurysms)^66^. The Early Treatment Diabetic Retinopathy Study (ETDRS) reported that early intervention with laser photocoagulation in DR appears associated with good long-term vision for patients, with 84% of patients demonstrating 20/40 or better in one of the eyes at long-term follow up^68^, highlighting the importance of early detection. Establishing a screening program for DR has shown significant benefits as in Iceland, one of the first countries to start such a program, which substantially decreased the prevalence of legal blindness in its diabetic population by 4.8 times^69^. Similar results were seen in the Newcastle District where a retina screening program decreased partial sightedness and blindness by more than two thirds^70^. Although screening programs for DR have proven to be highly effective at preventing blindness, they have yet to be implemented worldwide due to the various challenges including healthcare disparities.

Significant disparities exist in the access to eye care services worldwide with the majority of blindness (approximately 90%) found in developing countries where ophthalmic services are highly limited^71^. Many of these limitations, particularly access to screening and ophthalmic personnel, parallel similar situations seen during spaceflight. This mobile and efficient technology that has been adapted for spaceflight may address these barriers to optimal vision health and overall health of the community; recent advances have shown that retinal evaluation can help identify individuals at risk for cognitive and cardiovascular pathologies^72^. These findings further support the practical utility of cost-effective, mobile visual assessment technology for increased screening of retinal diseases. By building a comprehensive model of visual function for diabetic patients, the small footprint visual assessment and machine learning technology originally designed for SANS can help to address these challenges, providing low-cost, frequent screening for many at-risk individuals to reduce healthcare inequalities and the burden of debilitating diseases.

Innovative visual assessment technology using machine learning also has strong potential to prevent blindness in high-resource areas. Literature has shown that even in countries with strong healthcare infrastructures, over half of the causes of visual impairment are preventable (56%)^71,73^. Limburg and Keunen reported that the highest proportion of avoidable blindness in the Netherlands was seen in individuals with disabilities, followed by residents of nursing homes, illustrating how the lack of screening programs disproportionately affects high-risk individuals. Headset-based visual assessments with machine learning allow for more frequent screening for individuals with disabilities and reduce the need for in-person visits for immobile individuals. These implementations may improve the quality of life and economic independence within these populations.

Glaucoma and age-related macular degeneration (AMD) are also among the most common causes of preventable blindness worldwide, with AMD being most common cause of blindness in individuals over age 50 worldwide^64,74,75^ (Table 1). Open-angle glaucoma (OAG) is the most common type of glaucoma and often presents with subtle peripheral vision loss that can go undetected for years, leading to severe vision loss at an advanced stage without intervention^76^. OAG is often detected using three main measures: perimetry, optic nerve head morphology, and intraocular pressure (IOP)^77^. Although OAG can be effectively treated to attenuate progressive blindness, there are limited glaucoma screening programs to detect the subtle vision loss. Several reasons for this lack of population-based screening stem from unclear evidence of the longitudinal cost-effectiveness of screening and insufficient evidence for a simple and precise validated screening test^78,79^. IOP assessments are often performed with tonometry and optic nerve head morphology is often assessed with OCT, both typically requiring additional operators to perform the assessment^80,81^. The current gold standard for visual field perimetry testing is standard automated perimetry (SAP)^82^. However, SAP is stationary, has relatively large cost and space requirements^27^. This limits the areas where SAP can be available for individuals, particularly in underserved areas. Limited capacity in clinics have also been noted as a limitation and may lead to delays in follow-up appointments for glaucoma patients^27^. A potential solution to circumvent these barriers is to employ VR-based perimetry for high-risk individuals, providing relatively low-cost, more accessible monitoring for undetected peripheral vision loss from OAG. VR perimetry allows for portable, self-administration of visual field testing^83^. The asynchronous delivery of this portable assessment can increase accessibility in austere communities where SAP is physically unavailable or from delayed follow-up appointments. At-home VR-based perimetry has also been explored^84^ which may allow for more accessible testing in senior living communities where perimetry can be brought to community members. A current limitation is that it is still a relatively new technology that will likely require years of further research before becoming widely adopted. Although VR systems are much more cost-effective than SAP, individual home-based testing may not be cost-effective; however, the accessibility and delivery of VR-based perimetry in underserved areas may address barriers including high cost of stationary SAP and limited clinic capacity. A higher frequency in functional perimetry testing may allow for a more precise and personalized model of ocular function and structure relationship for OAG patients when combined with imaging modalities. Several risk factors for OAG also overlap with AMD, and VR vision assessment technology can serve as a particularly efficient screening modality for individuals that have these shared risk factors. Severe vision loss in AMD is primarily caused by the development of choroidal neovascularization (CNV)^74^. Multiple studies have demonstrated that early detection and intervention of CNV is essential to prevent vision loss^74^. FA has been deemed an option for early detection of CNV^74^. Thus, by employing VR-based metamorphopsia assessments and GANs for FA imaging for high-risk individuals, CNV can be rapidly detected and lead to timely intervention to prevent irreversible vision loss.

**Table 1:** Epidemiology, screening, treatments, and 20-year outlook for diabetic retinopathy, glaucoma, and age-related macular degeneration.

	Diabetic retinopathy	Glaucoma	Age-related macular degeneration
Epidemiology and Demographics most affected	An estimated 1 in 29 persons aged 40 or older in the US have diabetic retinopathy^86^. Diabetic retinopathy typically causes blindness during working-age years, resulting in substantial economic costs.	An estimated 70 million people worldwide have glaucoma, and it is the leading cause of irreversible blindness^28^. Risk factors include old age, family history of glaucoma, use of corticosteroids, elevated IOP, and African American ethnicity.	Estimated to account for 9% of all cases of blindness worldwide^87^. The main risk factors for AMD are genetic predisposition, age, and nicotine consumption.
Screening modalities and effectiveness	Ophthalmoscopy is the standard method to screen for diabetic retinopathy, with the earliest clinical sign being microaneurysms in the posterior pole. Fluorescein angiography is an invasive method to detect vascular changes in established diabetic retinopathy. OCT may provide additional information on the retinal layers^88^	Early diagnosis can be challenging due to insidious nature of disease progression. Recommended examination consists of a clinical history, tonometry, stereoscopic examination, and slit-lamp examination. Visual fields should also be examined a minimum of three times within the first year a diagnosis is made.	Initial symptoms of AMD often consist of central visual field loss or distorted vision. Diagnosis of AMD typically includes visual acuity, ophthalmic examination, examination of the macular layer with OCT, funduscopic evaluation with dilated pupils, and fluorescein angiography, if necessary^87^
Current treatments	Lifestyle modifications Anti-VEGF Intravitreal Injections Laser Photocoagulation	Eye drops, Trabeculectomy, Laser Trabeculoplasty, Minimally Invasive Glaucoma Surgery^28^	Depending on Wet/Dry Classification. Laser Photocoagulation. Anti-VEGF Intravitreal Injections
10–20 year outlook	The number of people with diabetic retinopathy worldwide is expected to reach 224 million by 2040^63^	In 2040, the number of people with glaucoma is expected to reach 111.8 million^64^	By 2040, 288 million people are expected to develop AMD^75^

Ultimately, the application of this mobile, low-cost visual assessment technology can allow for the detection of preventable visual impairments for underserved and/or high-risk patients that would otherwise not receive eye screening or ophthalmic care.

## Future outlook and summary

In the 1980’s, NASA developed laser radar (LADAR) technology for autonomous spacecraft docking in orbit. Today, this space-based innovation serves as integral precision, eye-tracking technology for LASIK, one of the most common ophthalmic surgeries^85^. The translational application of space technology continues to move forward in improving vision and quality of life on Earth. With projected outlook of increasing vision loss affecting millions in the coming decades, it is of utmost importance to apply cutting-edge technology to preserve vision and quality of life on Earth. The development of revolutionary VR and machine learning technology for SANS can help address many longstanding barriers to achieving healthy vision on Earth.

## References

[1] Lee AG (2020). Spaceflight associated neuro-ocular syndrome (SANS) and the neuro-ophthalmologic effects of microgravity: a review and an update. NPJ Microgravity.

[2] Patel ZS (2020). Red risks for a journey to the red planet: The highest priority human health risks for a mission to Mars. NPJ Microgravity.

[3] Mader TH (2011). Optic disc edema, globe flattening, choroidal folds, and hyperopic shifts observed in astronauts after long-duration space flight. Ophthalmology.

[4] Mader TH (2017). Persistent asymmetric optic disc swelling after long-duration space flight: implications for pathogenesis. J. Neuroophthalmol..

[5] Thomas H. et al. Persistent globe flattening in astronauts following long-duration spaceflight. *Neuro-Ophthalmology*, 10.1080/01658107.2020.1791189 (2020). 10.1080/01658107.2020.1791189 PMC7946045 33762785

[6] NASA. A Non-intrusive ocular monitoring framework to model ocular structure and functional changes due to long-term spaceflight (80NSSC20K1831). *NASA Life Sci. Data Archive* (2019).

[7] Ong, J. et al. A multi-modal visual assessment system for monitoring spaceflight associated neuro-ocular syndrome (SANS) during long duration spaceflight. *J. Vision* **22**, 10.1167/jov.22.3.6 (2022).

[8] NASA. NASA MEDB 1.10 Eye examinations. https://lsda.jsc.nasa.gov/lsda_data/document/Project/MRID/MEDB_1.10_1.10.1_Eye%20Examinations%2012_11_17_Project_13_27_17.pdf (2017).

[9] Ushakov. IB (2014). Main findings of psychophysiological studies in the Mars 500 experiment. Her. Russian Acad. Sci..

[10] Kintz NM, Palinkas LA (2016). Communication delays impact behavior and performance aboard the international space station. Aerosp. Med. Hum. Perform..

[11] Skelly, C. New Spinoff Publication Shares How NASA Innovations Benefit Life on Earth. *National Aeronautics and Space Administration* Space Tech, https://www.nasa.gov/press-release/new-spinoff-publication-shares-how-nasa-innovations-benefit-life-on-earth (2020).

[12] Nelson ES, Mulugeta L, Myers JG (2014). Microgravity-induced fluid shift and ophthalmic changes. Life (Basel).

[13] Wostyn P, De Deyn PP (2018). The “ocular glymphatic system”: an important missing piece in the puzzle of optic disc edema in astronauts?. Invest Ophthalmol. Vis. Sci..

[14] Killer HE, Jaggi GP, Flammer J, Miller NR, Huber AR (2006). The optic nerve: a new window into cerebrospinal fluid composition. Brain.

[15] Galdamez LA, Brunstetter TJ, Lee AG, Tarver WJ (2020). Origins of cerebral edema: implications for spaceflight-associated neuro-ocular syndrome. J. Neuroophthalmol..

[16] Strangman GE (2017). Increased cerebral blood volume pulsatility during head-down tilt with elevated carbon dioxide: the SPACECOT Study. J. Appl Physiol. (1985).

[17] Roberts DR (2017). Effects of spaceflight on astronaut brain structure as indicated on MRI. N. Engl. J. Med.

[18] Shinojima A, Kakeya I, Tada S (2018). Association of space flight with problems of the brain and eyes. JAMA Ophthalmol..

[19] Marshall-Goebel K, Damani R, Bershad EM (2019). Brain physiological response and adaptation during spaceflight. Neurosurgery.

[20] Yoon J, Drumright LN, van der Schaar M (2020). Anonymization through data synthesis using generative adversarial networks (ADS-GAN). IEEE J. Biomed. Health Inf..

[21] Ong, J. et al. Neuro-ophthalmic imaging and visual assessment technology for spaceflight associated neuro-ocular syndrome (SANS). *Surv. Ophthalmol*, 10.1016/j.survophthal.2022.04.004 (2022). 10.1016/j.survophthal.2022.04.004 35461882

[22] Gaskill, M. Nine Ways We Use AR and VR on the International Space Station. *NASA Space Station Research*, https://www.nasa.gov/mission_pages/station/research/news/nine-ways-we-use-ar-vr-on-iss (2021).

[23] Arvind H (2007). Dichoptic stimulation improves detection of glaucoma with multifocal visual evoked potentials. Invest Ophthalmol. Vis. Sci..

[24] Tsapakis S (2017). Visual field examination method using virtual reality glasses compared with the Humphrey perimeter. Clin. Ophthalmol..

[25] Sipatchin, A., Wahl, S. & Rifai, K. Eye-tracking for clinical ophthalmology with virtual reality (vr): a case study of the HTC vive pro eye’s usability. *Healthcare (Basel)* **9**, 10.3390/healthcare9020180 (2021). 10.3390/healthcare9020180 PMC7914806 33572072

[26] Clay, V., Konig, P. & Konig, S. Eye tracking in virtual reality. *J. Eye Mov. Res.* **12**, 10.16910/jemr.12.1.3 (2019). 10.16910/jemr.12.1.3 PMC7903250 33828721

[27] Stapelfeldt J, Kucur SS, Huber N, Hohn R, Sznitman R (2021). Virtual reality-based and conventional visual field examination comparison in healthy and glaucoma patients. Transl. Vis. Sci. Technol..

[28] Weinreb RN, Aung T, Medeiros FA (2014). The pathophysiology and treatment of glaucoma: a review. JAMA.

[29] Bennett CR, Bex PJ, Bauer CM, Merabet LB (2019). The assessment of visual function and functional vision. Semin Pediatr. Neurol..

[30] Ong J, Lee AG, Moss HE (2021). Head-down tilt bed rest studies as a terrestrial analog for spaceflight associated neuro-ocular syndrome. Front Neurol..

[31] Amanullah S (2017). The relationship between contrast sensitivity and retinal nerve fiber layer thickness in patients with glaucoma. Graefes Arch. Clin. Exp. Ophthalmol..

[32] Skalicky SE, Kong GY (2019). Novel means of clinical visual function testing among glaucoma patients, including virtual reality. J. Curr. Glaucoma Pr..

[33] Peters, M. Space station’s data rate increase supports future exploration. www.nasa.gov/feature/goddard/2019/data-rate-increase-on-the-international-space-station-supports-future-exploration **NASA** (2019).

[34] Savoy M (2020). IDx-DR for diabetic retinopathy screening. Am. Fam. Phys..

[35] Ting DSW (2019). Artificial intelligence and deep learning in ophthalmology. Br. J. Ophthalmol..

[36] Stein TP (2013). Weight, muscle and bone loss during space flight: another perspective. Eur. J. Appl. Physiol..

[37] Lee AY (2021). Multicenter, head-to-head, real-world validation study of seven automated artificial intelligence diabetic retinopathy screening systems. diabetes care 2021;44:XXXX-XXXX. Diabetes Care.

[38] Cheng PM, Malhi HS (2017). Transfer learning with convolutional neural networks for classification of abdominal ultrasound images. J. Digit Imaging.

[39] Morid MA, Borjali A, Del Fiol G (2021). A scoping review of transfer learning research on medical image analysis using ImageNet. Comput Biol. Med.

[40] Laurie SS (2021). Optic disc edema and chorioretinal folds develop during strict 6 degrees head-down tilt bed rest with or without artificial gravity. Physiol. Rep..

[41] He, K., Zhang, X., Ren, S. & Sun, J. Deep residual learning for image recognition. *2016 IEEE Conference on Computer Vision and Pattern Recognition (CVPR)*, 770-778, 10.1109/CVPR.2016.90 (2016).

[42] Dai, J., Li, Y., He, K. & Sun, J. R-FCN: Object Detection via Region-based Fully Convolutional Networks. *NIPS'16: Proceedings of the 30th International Conference on Neural Information Processing Systems*, 379–387, 10.5555/3157096.3157139 (2016).

[43] Schlemper J, Caballero J, Hajnal JV, Price AN, Rueckert D (2018). A deep cascade of convolutional neural networks for dynamic MR image reconstruction. IEEE Trans. Med. Imaging.

[44] Zhang K, Zuo W, Chen Y, Meng D, Zhang L (2017). Beyond a Gaussian Denoiser: residual learning of deep CNN for image denoising. IEEE Trans. Image Process.

[45] Fu, H., Xu, Y., Wong, D. & Liu, J. Retinal vessel segmentation via deep learning network and fully-connected conditional random fields. *IEEE International Symposium on Biomedical Imaging*, 698–701, 10.1109/ISBI.2016.7493362 (2016).

[46] Mitra A, Banerjee PS, Roy S, Roy S, Setua SK (2018). The region of interest localization for glaucoma analysis from retinal fundus image using deep learning. Comput. Methods Prog. Biomed..

[47] Di X (2017). Retinal hemorrhage detection by rule-based and machine learning approach. Annu Int Conf. IEEE Eng. Med Biol. Soc..

[48] Budai A, Bock R, Maier A, Hornegger J, Michelson G (2013). Robust vessel segmentation in fundus images. Int J. Biomed. Imaging.

[49] Vyas RJ (2020). Decreased vascular patterning in the retinas of astronaut crew members as new measure of ocular damage in spaceflight-associated neuro-ocular syndrome. Invest Ophthalmol. Vis. Sci..

[50] Khanal, A. & Estrada, R. Dynamic deep networks for retinal vessel segmentation. *Front. Comput. Sci.* **2**, 10.3389/fcomp.2020.00035 (2020).

[51] Teikari, P., Najjar, R. P., Schmetterer, L. & Milea, D. Embedded deep learning in ophthalmology: making ophthalmic imaging smarter. *Ther. Adv. Ophthalmol* **11**, 2515841419827172, 10.1177/2515841419827172 (2019). 10.1177/2515841419827172 PMC6425531 30911733

[52] Tan CC, Eswaran C (2011). Using autoencoders for mammogram compression. J. Med. Syst..

[53] Wang L (2019). A coarse-to-fine deep learning framework for optic disc segmentation in fundus images. Biomed. Signal Process Control.

[54] Ahn JM, Kim S, Ahn KS, Cho SH, Kim US (2019). Accuracy of machine learning for differentiation between optic neuropathies and pseudopapilledema. BMC Ophthalmol..

[55] Stenger, M. B. et al. Evidence report: risk of spaceflight associated neuro-ocular syndrome (SANS). *NASA Human Research Program Human Health Countermeasures Element*. https://humanresearchroadmap.nasa.gov/evidence/reports/SANS.pdf (2017).

[56] Tavakkoli A, Kamran SA, Hossain KF, Zuckerbrod SL (2020). A novel deep learning conditional generative adversarial network for producing angiography images from retinal fundus photographs. Sci. Rep..

[57] Wu M (2019). Geographic atrophy segmentation in SD-OCT images using synthesized fundus autofluorescence imaging. Comput Methods Prog. Biomed..

[58] You A, Kim JK, Ryu IH, Yoo TK (2022). Application of generative adversarial networks (GAN) for ophthalmology image domains: a survey. Eye Vis..

[59] Hajeb Mohammad Alipour S, Rabbani H, Akhlaghi MR (2012). Diabetic retinopathy grading by digital curvelet transform. Comput Math. Methods Med.

[60] Goodfellow, I. et al. Generative adversarial networks. *Advances in neural information processing systems* **27**, 10.1007/978-1-4842-3679-6_8 (2014).

[61] Christopher M (2020). Deep learning approaches predict glaucomatous visual field damage from OCT optic nerve head en face images and retinal nerve fiber layer thickness maps. Ophthalmology.

[62] Heidelberg. Heidelberg Engineering Introduces the GMPE Hood Glaucoma Report for SPECTRALIS OCT *Heidelberg Engineering Press,* https://www.heidelbergengineering.com/us/press-releases/heidelberg-engineering-introduces-the-gmpe-hood-glaucoma-report-for-spectralis-oct/ (2019).

[63] WHO. Strengthening diagnosis and treatment of Diabetic Retinopathy in SEA Region. *Regional Office for South-East Asia* World Health Organization, https://www.who.int/publications/i/item/9789290227946 (2020).

[64] Tham YC (2014). Global prevalence of glaucoma and projections of glaucoma burden through 2040: a systematic review and meta-analysis. Ophthalmology.

[65] WHO. Blindness and vision impairment. *World Health Organization Fact Sheet*, https://www.who.int/news-room/fact-sheets/detail/blindness-and-visual-impairment (2021).

[66] Ting DS, Cheung GC, Wong TY (2016). Diabetic retinopathy: global prevalence, major risk factors, screening practices and public health challenges: a review. Clin. Exp. Ophthalmol..

[67] Teo, Z. L. et al. Global prevalence of diabetic retinopathy and projection of burden through 2045: systematic review and meta-analysis. *Ophthalmology*, 10.1016/j.ophtha.2021.04.027 (2021). 10.1016/j.ophtha.2021.04.027 33940045

[68] Chew EY (2003). The long-term effects of laser photocoagulation treatment in patients with diabetic retinopathy: the early treatment diabetic retinopathy follow-up study. Ophthalmology.

[69] Stefansson E (2000). Screening and prevention of diabetic blindness. Acta Ophthalmol. Scand..

[70] Arun CS, Ngugi N, Lovelock L, Taylor R (2003). Effectiveness of screening in preventing blindness due to diabetic retinopathy. Diabet. Med.

[71] Taylor HR, Keeffe JE (2001). World blindness: a 21st century perspective. Br. J. Ophthalmol..

[72] Vujosevic S (2020). Screening for diabetic retinopathy: new perspectives and challenges. Lancet Diabetes Endocrinol..

[73] Limburg H, Keunen JE (2009). Blindness and low vision in The Netherlands from 2000 to 2020-modeling as a tool for focused intervention. Ophthalmic Epidemiol..

[74] Schwartz R, Loewenstein A (2015). Early detection of age related macular degeneration: current status. Int J. Retin. Vitreous.

[75] Dibas A, Yorio T (2016). Glucocorticoid therapy and ocular hypertension. Eur. J. Pharm..

[76] Schuster AK, Erb C, Hoffmann EM, Dietlein T, Pfeiffer N (2020). The diagnosis and treatment of glaucoma. Dtsch Arztebl Int.

[77] Stein JD, Khawaja AP, Weizer JS (2021). Glaucoma in adults-screening, diagnosis, and management: a review. JAMA.

[78] Taylor H (2011). Glaucoma screening in the real world. Ophthalmology.

[79] Hamid, S., Desai, P., Hysi, P., Burr, J. M. & Khawaja, A. P. Population screening for glaucoma in UK: current recommendations and future directions. *Eye*, 10.1038/s41433-021-01687-8 (2021). 10.1038/s41433-021-01687-8 PMC8873198 34345031

[80] Resch H (2018). Optic nerve head morphology in primary open-angle glaucoma and nonarteritic anterior ischaemic optic neuropathy measured with spectral domain optical coherence tomography. Acta Ophthalmol..

[81] Tonnu PA (2005). A comparison of four methods of tonometry: method agreement and interobserver variability. Br. J. Ophthalmol..

[82] Alencar LM, Medeiros FA (2011). The role of standard automated perimetry and newer functional methods for glaucoma diagnosis and follow-up. Indian J. Ophthalmol..

[83] Montelongo M, Gonzalez A, Morgenstern F, Donahue SP, Groth SL (2021). A virtual reality-based automated perimeter, device, and pilot study. Transl. Vis. Sci. Technol..

[84] Deiner MS, Damato BE, Ou Y (2020). Implementing and monitoring at-home virtual reality oculo-kinetic perimetry during COVID-19. Ophthalmology.

[85] NASA. The right track for vision correction *NASA SpinOff*, https://spinoff.nasa.gov/spinoff2003/hm_1.html (2003).

[86] Kempen JH (2004). The prevalence of diabetic retinopathy among adults in the United States. Arch. Ophthalmol..

[87] Stahl A (2020). The diagnosis and treatment of age-related macular degeneration. Dtsch Arztebl Int.

[88] Corcostegui B (2017). Update on diagnosis and treatment of diabetic retinopathy: a consensus guideline of the working group of ocular health (Spanish Society of Diabetes and Spanish Vitreous and Retina Society.. J. Ophthalmol..

